# Physical and Mental Effects of Foot Baths Among Women in Labor: Protocol for a Pre-Post Test Experimental Design

**DOI:** 10.2196/39985

**Published:** 2023-01-18

**Authors:** Naoko Hikita, Ritsuko Iso, Kiyoko Mizuhata, Akemi Isoyama, Ayumi Kobayashi, Rika Muroi

**Affiliations:** 1 Department of Health Sciences Graduate School of Medical Sciences Kyushu University Fukuoka Japan; 2 Graduate Program of Midwifery Dokkyo Medical University Mibu-machi, Shimotsuga-gun, Tochigi Japan; 3 Kamitsuga General Hospital Kanuma-shi, Tochigi Japan

**Keywords:** foot bath, women in labor, salivary cortisol, pregnant, pregnancy, alternative medicine, complementary medicine

## Abstract

**Background:**

Foot baths are used in complementary and alternative therapy to improve the duration and quality of sleep and reduce tension, anxiety, fatigue, and confusion. They are also known to improve the frequency of labor contractions and to increase their duration in women; thus, they are commonly used by midwives in clinical settings in Japan. However, the physical and mental effects of foot baths during labor are unknown.

**Objective:**

This study aims to assess the physical and mental effects of foot baths based on biomarker levels and self-administered questionnaires.

**Methods:**

A single-arm pre-post test trial design is being used in this study, and the study is being conducted at a general hospital in Tochigi Prefecture, Japan. The target study population is women in the first stage of labor, the phase when the uterus starts to contract and when the cervix dilates to 10 cm, or those undergoing labor induction. Participants who meet the eligibility criteria are recruited, and written informed consent is obtained from them. They are asked to answer the questionnaire and to collect 1.5 mL of saliva in 2 microtubes each, before and after the intervention. The intervention is foot baths for 15-20 minutes using a foot bath device. Data on delivery, such as gestational age, gravidity, parity, diagnosis following the last vaginal examination, and presence or absence of membrane rupture, are retrieved from the medical records. The primary outcomes are salivary cortisol levels before and after the foot baths. The secondary outcomes are levels of relaxation and comfort, labor pain, body warmth, vital signs, and interval of labor pain before and after the foot baths, which are assessed using a numerical rating scale. A paired *t* test or Wilcoxon signed-rank test will be performed to compare the data for salivary cortisol levels and numerical rating scale scores.

**Results:**

Data collection started on April 1, 2022. As of October 2022, we had enrolled 10 participants. Because of the COVID-19 pandemic in Japan, it is difficult for medical personnel to freely interact with women in labor until the results of the COVID-19 polymerase chain reaction test are available in the research facility, complicating the recruitment process.

**Conclusions:**

This is the first prospective study to assess the effects of foot baths using a biomarker during the first stage of labor. The findings on the effects of foot baths on women in labor will provide novel insights that may improve the outcomes of delivery. A randomized controlled trial to investigate the effects of foot baths to obtain robust evidence should be conducted in the future.

**Trial Registration:**

University Hospital Medical Information Network-Clinical Trial Registry UMIN000046539; https://tinyurl.com/2wwj7dns

**International Registered Report Identifier (IRRID):**

DERR1-10.2196/39985

## Introduction

### Background

Foot baths are used for cleaning the feet and are useful in complementary and alternative therapy. Foot baths improve the duration and quality of sleep among postmenopausal and older adults [1-4], decrease low back pain in pregnant women [5], and have psychological effects, such as reduced tension, anxiety, fatigue, and confusion in women [6,7]. They are also known to be effective in decreasing edema of the lower limbs by improving blood circulation due to blood vessel dilation [8].

Studies evaluating the effect of foot baths in the first stage of labor reported that they improve the frequency of labor contractions in women with slow labor [9] and increase the duration of labor contractions in primiparous women [10]. Thus, foot baths may show effects on labor induction.

The epidural labor rate has been increasing in Japan (4.6% in 2014, 5.5% in 2015, and 6.1% in 2016) [11], although it remains lower than that in European or American countries [11-15]. One reason for the low rate of epidural labor in Japan is the low number of anesthetists and obstetricians in the country, affecting their availability for all cases. Therefore, several nonmedical methods are used for labor pain relief in clinical settings, including hot bath therapy, reflexology, aroma therapy, breathing, and massage [15-17]. Although previous studies have reported the psychological and labor-inducing effects of foot baths [6,7,9,10], the sample size of some of these studies was small, and others did not include women in labor. Furthermore, the physical and mental effects of foot baths during labor, such as the effects on relieving labor pain, improving relaxation, or decreasing stress, remain unknown, and no study has investigated the effect of foot baths on women in labor using biomarkers. Cortisol is a hormone secreted by the suprarenal cortex and is widely used as a biomarker to measure stress levels. If foot baths are shown to reduce stress in women in labor, they can be recommended for the nonmedical care of women in labor. Therefore, investigating the physical and mental effects of foot baths on women in labor, especially their pain-relieving effects, can facilitate mental relief and a satisfactory childbirth experience for women in labor. Foot baths are commonly used by midwives in clinical settings in Japan and are expected to have psychological and labor-inducing effects.

### Study Aims

This study aims to investigate the physical and mental effects of foot baths using a biomarker and self-administered questionnaire.

## Methods

### Study Design

We are conducting a single-arm, pre-post test trial to evaluate the effectiveness of foot baths.

### Study Setting and Population

The study is being conducted at a general hospital in Tochigi Prefecture, Japan. The inclusion criteria are women in the first stage of labor or those undergoing labor induction. The first stage of labor is the phase when a pregnant woman’s uterus starts to contract and they feel persistent contractions and relaxation, and the cervix dilates to 10 cm. Women who cannot complete the questionnaire or cannot participate in the study because of labor pain or those who are judged to have difficulty participating in the study by midwives because of low literacy of Japanese or due to mental illnesses are excluded from recruitment.

### Intervention Description

**Figure 1:**
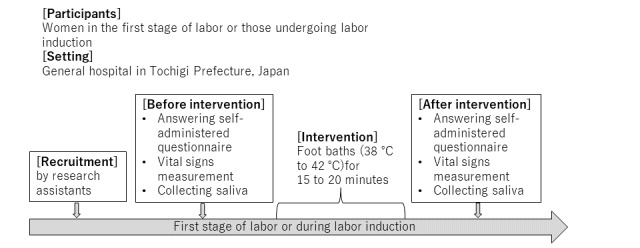
Protocol of the study.

This is a pre-post test trial; therefore, all participants are to receive the intervention. In this study, a foot bath is defined as the immersion of feet up to the ankle in hot water, set to a temperature that feels comfortable to the participants (38 °C to 42 °C) for 15-20 minutes (Figure 1). During the foot bath, other stimuli, such as pushing acupoints or massage, are avoided, because a meta-analysis has shown the effects of acupressure on labor duration [18]; this point was explained to all research assistants before starting this study. Participants receive the foot bath intervention only once, because midwives usually provide foot baths for women in labor only once in clinical settings; therefore, we aim to evaluate the effects of a single foot bath intervention. The intervention is discontinued if delivery progresses rapidly and if the midwives consider it difficult to continue.

### Data Collection

#### Variables

Data collection is being undertaken by research assistants, who are midwives working in the hospital and on duty, as assigned by the midwife in charge.

Data are collected using a self-administered questionnaire, laboratory assay for the biomarker, anthropometric measurements, and medical records. The self-administered questionnaire was adapted from those developed in previous studies that assessed the effects of foot baths [7,19-23]. Participants are asked to answer a questionnaire evaluating sociodemographic data, such as age, marital status (married, single, divorced, or widowed), education level (junior high or high school; vocational school, junior college, or technical college; and university or graduate school), and occupation (employee, public servant, self-employed, part-time worker, unemployed or housewife, and others). Previous studies used a 10 cm ruler to assess the comfort or feeling of body warmth [19,24], with 0 cm meaning “not at all” and 10 cm meaning “extremely so”; thus, in this study, a numerical rating scale (NRS), ranging from 0 to 10, is being used to collect data on the levels of labor pain and comfort, relaxation, and degree of warmth of the legs and entire body. Participants are asked to provide 1.5 mL of saliva in 2 microtubes before and after the foot bath. Vital signs, such as blood pressure, body temperature, pulse rate, and respiratory rate, are measured by research assistants before and after the foot bath. Data on delivery (gestational age, gravidity, parity, diagnosis following the last vaginal examination [such as dilation, station, effacement, consistency, and position], presence or absence of membrane rupture, use of uterine contraction agents, and estimated fetal body weight) are retrieved from the medical records by the research assistants and entered in the questionnaire.

The primary outcome of this study is salivary cortisol levels before and after the foot bath. The secondary outcomes are levels of labor pain, comfort and relaxation, and leg and body warmth, assessed using NRS scores, vital signs, and intervals of labor pain before and after the foot bath.

#### Data Collection Methods

We asked some experienced midwives working at the hospital to assist in this study as research assistants. Before study initiation, instructions on data collection and intervention were provided and explained to the research assistants.

The research assistants recruit participants for the study and explain the purpose and contents of the study using an instruction sheet. After obtaining oral consent, written informed consent is obtained.

The research assistants provide the participants with the self-administered questionnaire along with a clipboard and pen. Participants are asked to complete the self-administered questionnaire. After completion, the midwives measure the participants’ vital signs (blood pressure, body temperature, pulse rate, and respiratory rate) and the interval and duration of labor pain.

They also request participants to provide 1.5 mL of saliva in 2 microtubes each. After saliva collection, the specimens are stored at −20 °C until analysis.

The research assistants provide foot baths to participants using a foot bath device (Hietori-kun Micon Premium, FB-C80, Koyo Co, Ltd, Gifu, Japan). The temperature of the water is set to 38 °C to 42 °C, as desired by the participants. AS mentioned, during the foot bath, the research assistants avoid applying pressure on acupoints. The participants are allowed to soak their feet in the bath quietly for 15-20 minutes.

After the foot bath, the research assistants provide the participants with a self-administered questionnaire with a clipboard and pen. After completing the questionnaire, they measure the vital signs (blood pressure, body temperature, pulse rate, and respiratory rate) and the interval and duration of labor pain.

After saliva collection, the specimens are stored at −20 °C until analysis. If the participants have difficulty providing saliva because of severe labor pain, only responding to the self-administered questionnaire and the measurement of the vital signs is allowed.

After recruitment, all salivary cortisol level measurements are conducted at Yanaihara Institute Inc, Shizuoka, Japan.

We will continue to recruit participants until the target sample size is attained, and we may consider adding another hospital as a study setting in case the recruitment does not progress favorably because of the COVID-19 pandemic. It is difficult for medical personnel to freely interact with women in labor until the results of the COVID-19 polymerase chain reaction test are available, complicating the recruitment process.

Data are double-checked during entry, and the data ranges are checked to ensure data quality. The collected data and questionnaires are stored in a locked cupboard.

### Sample Size Calculation

According to a previous study that investigated differences in salivary cortisol levels before and after foot baths [20], the sample size was calculated based on the following considerations: effect size (Cohen *d*)=0.4, α (2-tailed)=.05, and *β*=.80. We estimated the sample size using a two-tailed *t* test to be equal to 100.

### Patient and Public Involvement

Neither patients nor the public are involved in the design, conduct, reporting, or dissemination of the study.

### Data Analysis

Categorical variables, such as marital status, education level, and occupation, will be presented as n (%), and continuous variables (salivary cortisol levels; NRS scores for labor pain, comfort, and relaxation; and vital signs, such as blood pressure, body temperature, pulse rate, and respiratory rate) will be presented as mean (SD) if normally distributed or as medians and interquartile ranges if nonnormally distributed. We will perform paired *t* tests for continuous variables if the observations are normally distributed, and if they are not, we will perform the Wilcoxon signed-rank test to compare data before and after foot baths. Furthermore, we will divide the participants into 2 groups, nullipara and multipara, for sensitivity analysis. All missing pretest or posttest data for the main outcome will be excluded from the analysis.

All data will be analyzed using SPSS Statistics for Windows (version 28.0; IBM Corp). Two-tailed *P* values of <.05 will be considered statistically significant.

### Harms

In this study, foot bath devices with a constant temperature of hot water are used. When preparing hot water, the midwives check the temperature using a thermometer; however, the possibility of burns to the feet cannot be ruled out. Moreover, water spills on the floor may cause falls. In case of accidents, the midwives report to the obstetrician of the maternity ward and arrange for a doctor’s visit. The cost of medical treatment is to be covered by the participant’s health insurance coverage.

### Ethics Approval and Consent to Participate

This study was approved by the Research Ethics Committee of the School of Nursing, Dokkyo Medical University, Japan (No. Nursing 03019) and the Research Ethics Committee of Kamitsuga General Hospital (No. 2021-008). The research assistants explain the purpose and contents of the study using an instruction sheet. Participation is voluntary. The possibility of harm and financial burden in case of accidents is explained to the participants. Written informed consent is obtained from all participants. Participants’ identifying information, such as their name, home address, and date of birth, is handled only by the hospital staff, and the information is never taken out of the research facility. All data will be analyzed anonymously, and participants’ other identifying information will be removed before analysis. The results of this study will be disseminated to scientific meetings and journals.

### Trial Registration

This study was registered in the University Hospital Medical Information Network-Clinical Trial Registry system (UMIN000046539) on January 17, 2022.

## Results

Data collection started on April 1, 2022. As of October 2022, we had enrolled 10 participants. Because of the COVID-19 pandemic in Japan, it is difficult for medical personnel to freely interact with women in labor until the results of the COVID-19 polymerase chain reaction test are available in the research facility, complicating the recruitment process. Moreover, the number of medical personnel who can engage in the research decreased in the research facility because some staff were infected by COVID-19; thus, recruitment is currently not progressing.

## Discussion

### Principal Findings

This is the first prospective study that will assess the effects of foot baths using a biomarker (salivary cortisol) in the first stage of labor. Though foot baths are commonly used by midwives in clinical settings in Japan and are expected to have psychological and labor-inducing effects, psychological effects using biomarkers have not been shown. Participant recruitment started on April 2022, but because of the COVID-19 pandemic in Japan, recruitment is currently not progressing.

Though several studies have reported the physical and mental effects of foot baths, they did not include women in labor as participants [1-8]. Previous studies that targeted women in labor [9,10,25] had several limitations, which are as follows: had a small sample size [9], focused only on the progress of labor effects of foot baths [9,10], and used the visual analog scale to assess anxiety [25]. In clinical settings, midwives sometimes apply pressure to acupoints during foot baths, and they use aroma as part of a midwife’s care during the first stage of labor. A meta-analysis has shown the effects of acupressure on labor duration [18], and aroma increased the effects of foot baths [25,26]. Therefore, in this study, we are implementing a simple foot bath without any acupressure or aroma, and the research assistants follow the study protocols because the effects of acupressure and aroma are widely known to midwives in Japan.

In Japan, the rate of epidural labor has been increasing recently [11]. Midwives traditionally implement nonpharmacological care in Japan, and foot baths are a form of such care. However, the physical and mental effects of foot baths have not been fully investigated, especially using biomarkers. Therefore, the findings for the effects of foot baths on women in labor will provide a novel perspective that will be useful worldwide, especially in developing countries where pharmacological care is difficult to access.

The appropriateness of the methodology of this study should be discussed. In this study, the outcomes are measured using salivary cortisol and an NRS for the levels of labor pain and comfort, relaxation, and the degree of warmth of the legs and the entire body. Samples are collected during labor, and therefore, collecting saliva or answering the questionnaire may trigger pain or stress for some participants. However, scoring NRS is not time-consuming, and several studies have used visual analog scales to assess comfort or warmth [19,20,24]; thus, it may be better than using a mental scale. The information about the participants’ characteristics, such as marital status, is obtained in this study because participants’ characteristics may affect their mental condition under the stress of labor. Moreover, this study is conducted in only 1 prefecture in Japan; thus, participants’ characteristics may be biased, and the results should be interpreted with caution.

Furthermore, we need to mention the potential physical harm from foot baths. Foot baths are one of the popular midwifery interventions in Japan due to their psychological and labor-inducing effects. Therefore, most midwives understand the necessary precautions, such as controlling the temperature of hot water and avoiding water spills on the floor. Nevertheless, in this study, we use foot bath devices to maintain the correct temperature of the hot water, although we cannot fully rule out the possibility of burns to the feet.

Moreover, this study uses a single-arm, pre-post test trial design; thus, the interpretation of the study findings requires some attention. With such a design, the results will only show within-individual differences. To obtain robust evidence on the effects of foot baths during labor, a randomized controlled trial should be conducted in the future.

### Strengths and Limitations

The strength of this study is that this is the first study that assesses the effects of foot baths during the first stage of labor using a biomarker (salivary cortisol). Though several previous studies have reported the effects of foot baths, few have evaluated them using biomarkers; moreover, there are no studies using biomarkers among women in childbirth.

Nevertheless, this study has some limitations. First, the design of this study is a single-arm pre-post test trial; thus, it is difficult to judge the effects of foot baths. To obtain robust evidence of the effects of foot baths, randomized controlled trials should be conducted in the future. Second, because of the COVID-19 pandemic, participant recruitment has not progressed favorably. Therefore, adding another hospital as a study setting has to be considered.

### Conclusion

This is the first prospective study that will assess the effects of foot baths using a biomarker (salivary cortisol) in the first stage of labor. Though participant recruitment has not progressed favorably because of the COVID-19 pandemic, if foot baths are shown to reduce stress in women in labor, they can be recommended for the nonmedical care of women in labor. A randomized controlled trial to investigate the effects of foot baths to obtain robust evidence should be conducted in the future.

## Abbreviations

NRS: numerical rating scale

## Availability of Data and Materials

Data sharing is not applicable to this article as no data sets have yet been generated or analyzed for the study.
